# Impact of CT-assessed sarcopenia on the severity of odontogenic deep neck infections: a retrospective cohort study

**DOI:** 10.1007/s10266-025-01204-3

**Published:** 2025-09-17

**Authors:** Shogo Kikuta, Eiji Iwata, Yohei Takeshita, Chizuru Kobayashi, Hiroki Kimura, Yuki Kinisada, Akira Tachibana, Jingo Kusukawa, Masaya Akashi, Soichiro Ibaragi

**Affiliations:** 1 Dental and Oral Medical Center, Kurume University School of Medicine, Kurume, Japan; 2 Department of Oral and Maxillofacial Surgery, Dentistry and Pharmaceutical Sciences, Okayama University Graduate School of Medicine, 2-5-1 Shikata-cho, Kitaku, Okayama 700-8525 Japan; 3 Department of Oral and Maxillofacial Radiology, Dentistry and Pharmaceutical Sciences, Okayama University Graduate School of Medicine, Okayama, Japan; 4 Department of Oral and Maxillofacial Surgery, Kakogawa Central City Hospital, Kakogawa, Japan; 5 Department of Oral and Maxillofacial Surgery, Kobe University Graduate School of Medicine, Kobe, Japan

**Keywords:** CT-assessed sarcopenia, Odontogenic deep neck infections, Severity, Hospitalization duration, Skeletal muscle index

## Abstract

Sarcopenia is increasingly recognized as a key predictor of adverse health outcomes. This study aimed to evaluate the impact of computed tomography-assessed sarcopenia (CT–SP) on the clinical severity and hospitalization duration of odontogenic deep neck infections (DNIs). Total of 119 patients admitted for odontogenic DNI treatment were included. Patients were divided into two groups by DNI clinical severity (severe or mild) and the patients' characteristics, including CT–SP based on skeletal muscle index (SMI), were compared between two groups. Multivariable logistic regression analysis was performed to identify independent risk factors for severe DNI. The correlation between SMI and hospitalization duration was assessed using Spearman’s rank correlation coefficient. Of the 119 patients, 60 (50.4%) presented with severe DNIs, including deep neck abscesses and necrotizing soft tissue infections. After adjusting for potential confounders, multivariable analysis identified CT–SP as the sole independent risk factor associated with severe DNI (Odds Ratio = 3.04; 95% Confidence Interval, 1.20–7.71; *p* = 0.019). Furthermore, SMI demonstrated a significant, weak negative correlation with the hospitalization duration (*r* = − 0.331, *p* < 0.001). CT–SP is a powerful, independent risk factor associated with severity in patients with odontogenic DNIs. This finding underscores the critical role of systemic host factors in the clinical course of maxillofacial infections and highlights the potential of opportunistic CT screening as a factor to consider in risk stratification in this vulnerable population.

## Introduction

Sarcopenia, a progressive and generalized skeletal muscle disorder defined by the loss of muscle mass, strength, and physical performance, is increasingly recognized as a critical predictor of adverse health outcomes, particularly in geriatric and chronically ill populations [1, 2]. It contributes not only to frailty and functional decline but also to increased vulnerability to infectious diseases [3]. The underlying pathophysiology of sarcopenia—encompassing chronic inflammation, hormonal dysregulation, and mitochondrial dysfunction—is thought to directly compromise immune competence and wound healing capabilities [4]. Collectively, these factors render sarcopenic individuals more vulnerable to acquiring infections and experiencing a more severe clinical course [5].

Odontogenic deep neck infections (DNIs) are severe bacterial infections that arise from dental or periodontal foci and extend into the deep fascial spaces of the neck [6]. Despite significant advances in antimicrobial therapy and diagnostic imaging, DNIs continue to pose a serious clinical challenge due to their potential for rapid progression and life-threatening complications, such as airway compromise, mediastinitis, and sepsis [7]. Clinical outcomes largely depend on rapid diagnosis, effective antibiotic treatment, and, when necessary, incisional drainage [7]. However, it is recognized that the clinical course and ultimate prognosis of these infections are profoundly influenced by host-specific factors, including age, nutritional status, and underlying comorbidities [8, 9].

While compromised host conditions, such as diabetes mellitus, are well-established risk factors for poor outcomes in odontogenic DNIs [8, 9], the specific impact of sarcopenia on the disease severity and course remains inadequately explored. Recent studies suggest that sarcopenia is an independent predictor of adverse outcomes across various infectious and surgical contexts, including pneumonia, sepsis, and postoperative recovery [10, 11]. Given the physiological burden of odontogenic DNIs and their potential for rapid deterioration, the presence of sarcopenia could be a critical determinant of the clinical trajectory. Sarcopenia is conventionally diagnosed by measuring skeletal muscle mass at the third lumbar vertebra (L3) on CT scans [12], an imaging study not routinely performed for maxillofacial conditions, such as DNIs and oral cancer. To overcome this, recent studies have validated the assessment of skeletal muscle mass at the third cervical vertebra (C3) as a reliable alternative in patients with oral cancer [13–15]. Therefore, leveraging this adapted methodology, this study aimed to investigate the relationship between low skeletal muscle mass—as quantified by CT-based assessment at C3 (CT-assessed sarcopenia; CT–SP)—and the severity and hospitalization duration of odontogenic DNIs.

## Patients and methods

### Patients

This study represents a sub-analysis of a larger investigation into 'Multicenter collaborative study on the pathophysiology of deep neck infections' conducted under the auspices of Kurume University. The present analysis focuses specifically on the relationship between CT–SP and disease severity. Other aspects of this cohort, such as inflammatory pathways and nutritional markers, have been or will be reported separately [9]. The present analysis focuses specifically on a retrospective cohort of patients treated at Kakogawa Central City Hospital, one of the participating institutions, between January 2017 and December 2022.

Inclusion criteria encompassed both male and female patients over 18 years who had been hospitalized for the treatment of odontogenic DNIs more than 48 h and had received intravenous antibiotics. The criteria for hospitalization were based on clinical signs such as skin erythema, dysphagia, difficulty eating, and increased inflammatory markers detected in blood tests. Exclusion criteria included patients who refused participation following the study's publication. Populations were divided into two groups, i.e., severe and mild DNIs. For the purposes of this analysis, we defined severe DNIs as pathologies requiring urgent surgical intervention, namely, deep neck abscesses and necrotizing soft tissue infections (NSTIs). The deep neck abscess was diagnosed whether abscess formation in the deep neck spaces by CT, while the definition of NSTI was based on previous studies [8, 9, 16]. In simple terms, the diagnosis of NSTI was established using the criteria defined by Fisher [17] and Mathieu [18], and further validated through evidence of gas production in CT imaging, findings observed during surgery, and histopathological analysis. This classification is based on their significantly elevated risk for life-threatening complications, such as airway compromise and sepsis, thereby representing a clinically homogenous group of high-acuity patients. Mild DNIs consist of cellulitis and cellulitis with superficial abscess formation localized to a single region without deep anatomical space involvement, as in previous studies [19, 20]. If an abscess had formed, incisional drainage was performed urgently, and the drained pus or necrotic tissue was sent for bacterial culture.

### Computed tomography assessment of sarcopenia (CT–SP)

**Fig. 1:**
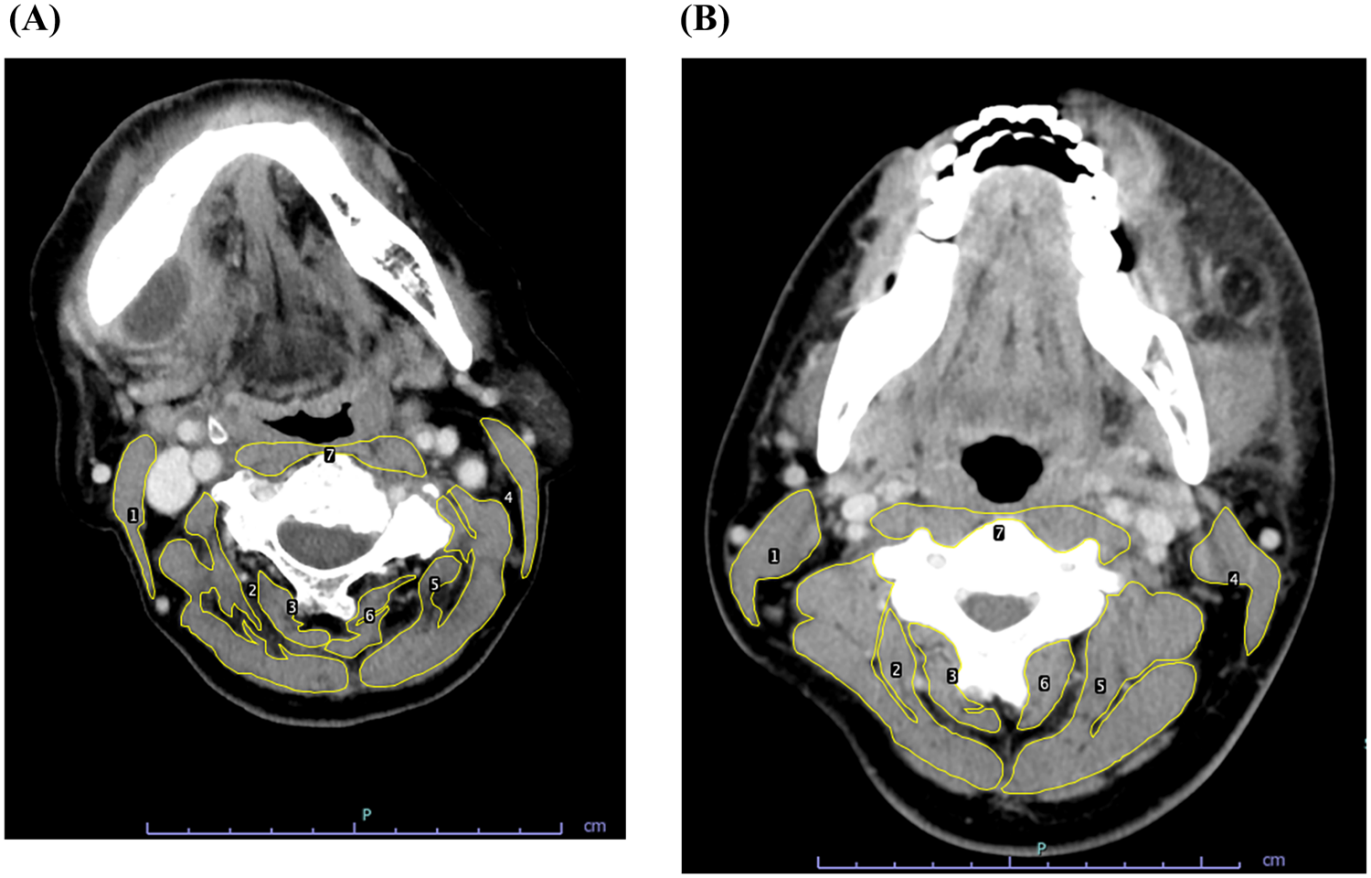
Measurements of computed tomography (CT)-assessed sarcopenia. The total cross-sectional areas (CSA) of the sternocleidomastoid and paravertebral muscles were calculated by preoperative cervical CT using the open-source software ImageJ. CT slices of a patient in severe DNIs group (**A**) and a patient in mild DNIs group (**B**). The area surrounded by the yellow line is the muscle to be measured. Numbers indicate muscle blocks. No.1 and 4 are sternocleidomastoid muscles. No. 2, 3, 5, 6, and 7 are paravertebral muscles

In all patients, CT data at the time of admission were evaluated. Given that abdominopelvic CT is not part of the standard diagnostic workup for this patient population, we employed the validated C3 vertebra method to assess skeletal muscle mass [14, 15]. This approach permits a pragmatic, opportunistic assessment of low skeletal muscle using existing cervical CT images without incurring additional radiation exposure or healthcare costs. Following previous research, we adopted the slice, where the transverse and spinous processes of the third cervical vertebra were most clearly visible [14, 15]. All images were analyzed by an oral radiologist (YT) with more than 10 years of experience using the open-source software ImageJ (Fig. 1).

The total cross-sectional area (CSA) in cm^2^ of the sternocleidomastoid and paravertebral muscles was measured at the C3 level. From this, the CSA at the L3 was estimated using the following previously validated formula:

$${\mathrm{L}}3\,{\mathrm{CSA}}\,\left( {{\mathrm{cm}}^{2} } \right)\, = \,27.304 + 1.363 \times {\mathrm{C}}3\,{\mathrm{CSA}}\,\left( {{\mathrm{cm}}^{2} } \right) - 0.671 \times \,{\mathrm{age}} + 0.640 \times {\mathrm{weight}}\,\left( {{\mathrm{kg}}} \right) + 26.442 \times {\mathrm{sex}}\,(where\,sex\,was\,coded\,as\,1\,for\,female\,and\,2\,for\,male)$$

The skeletal muscle index (SMI) was then calculated by normalizing the estimated L3 CSA by the square of the patient's height (m^2^), as follows [14, 15]:

$${\mathrm{SMI}}\,\left( {{\mathrm{cm}}^{2} /{\mathrm{m}}^{2} } \right) = {\mathrm{L}}3\,{\mathrm{CSA}}\,\left( {{\mathrm{cm}}^{2} } \right)/\left( {{\mathrm{height}}\,\left( {\mathrm{m}} \right)} \right)^{2}$$

Finally, CT–SP was defined using established, sex-specific SMI cutoffs for Japanese populations: < 43.2 cm^2^/m^2^ for males and < 34.6 cm^2^/m^2^ for females [14, 15].

### Data collection

The following clinical and laboratory data were retrospectively collected from each patient's electronic medical record: demographic data (age, sex, body mass index [BMI]); clinical status (World Health Organization Performance Status [WHO-PS], presence of a compromised host condition, smoking history); an imaging-based sarcopenia assessment (CT–SP); serum albumin (Alb) in blood test, and hospitalization duration). BMI was calculated as weight (kg) divided by the square of the height (m^2^) [21]. The WHO-PS was graded on a scale from 0 (fully active) to 5 (death), as previously described [22]. The compromised host was defined as a patient with pre-existing conditions known to affect immune function, such as diabetes mellitus, chronic kidney failure, or rheumatoid arthritis. All baseline radiographic and laboratory assessments were based on data obtained at the time of admission.

All CT imaging was performed using either a 64-slice system (Aquilion 64; Canon Medical Systems Corp., Tochigi, Japan) or a 128-slice system (SOMATOM Definition Flash; Siemens, Munich, Germany). Scans were acquired under standard head and neck protocols with automatic exposure control (120 kV; 1–5 mm slice thickness). Multiplanar reconstruction (MPR) images were generated from the source data using Digital Imaging and Communications in Medicine (DICOM) viewer software (Zyostation2 Type H, Ziosoft Inc., Tokyo, Japan). When required, one of the following contrast agents was administered: Iomeron 300 (Eisai, Tokyo, Japan), Iopamirdol 370 (Hikari Pharmaceutical, Tokyo, Japan), or Omnipaque 300 (GE Healthcare, Chicago, IL, USA).

### Ethical approval

This study was conducted in strict adherence to the principles of the Declaration of Helsinki (as amended in Fortaleza, 2013). The research protocol was approved by the Institutional Review Board (IRB) of Kurume University Hospital (Approval No. 23122). Given the retrospective nature of the study, the IRB granted a waiver of patient-specific informed consent. In lieu of individual consent, and as stipulated by the IRB, information regarding the study was publicly disclosed on the official website of our institution, providing patients with the opportunity to opt-out of having their data used for research purposes.

### Statistical analysis

All statistical analyses were conducted using SPSS Statistics for Windows, version 26.0 (IBM Corp., Armonk, NY, USA) and Ekuseru–Toukei 2016 software (Social Survey Research Information Co. Ltd., Tokyo, Japan). A two-sided *p* value < 0.05 was considered statistically significant for all analyses. Univariate analyses were first performed to assess the association of each variable with the presence of severe DNIs. The Mann–Whitney *U* test was used for ordinal variables, while the chi-square test or Fisher’s exact test was applied for categorical variables. The correlation between key continuous variables was evaluated using Spearman’s rank correlation coefficient (ρ). Subsequently, a multivariable logistic regression model was constructed using the forced-entry method. All variables found to be significantly associated with severe DNIs in the univariate analysis were included in the model. However, to avoid issues with multicollinearity, variables that are constituent components of the Skeletal Muscle Index (SMI)—namely, age, sex, and BMI—were excluded from the model. Furthermore, variables with a substantial proportion of missing data, specifically serum albumin (26.1% missing), were also excluded to ensure the statistical stability and robustness of the final model. Prior to finalizing the model, multicollinearity among the independent variables was assessed using the Variance Inflation Factor (VIF). After model construction, a goodness-of-fit test was performed to evaluate model calibration. The results of the logistic regression are presented as odds ratios (ORs) with their corresponding 95% confidence intervals (CIs).

## Results

### Patient characteristics and univariate analysis

Of the 119 patients included in the study, 60 (50.4%) were classified into the severe DNI group (Fig. 1). The baseline characteristics and the results of the univariate comparison between the severe and mild DNI groups are summarized in Table 1. In the univariate analysis, patients in the severe DNI group were significantly older (*p* = 0.008), more frequently had a compromised host status (*p* = 0.033), and showed a higher prevalence of CT–SP (*p* = 0.006). Correspondingly, the SMI was significantly lower in the severe DNI group compared to the mild DNI group (median: 37.1 vs. 44.3, respectively; *p* = 0.020). Of course, inflammatory markers, particularly the CRP level, were significantly elevated in the severe DNI group (*p* < 0.001) (data not shown). In contrast, Alb levels, an indicator of nutritional status, were significantly lower in this group (*p* < 0.001).

**Table 1:** Comparison between severe and mild DNIs groups

Variable		Severe DNIs group (*n* = 60)	Mild DNIs group (*n* = 59)	*p* value
Age (years)	Median (range)	65.5 (19–93)	52.0 (18–89)	0.008*^b^
Sex	Male	28 (46.7%)	29 (49.2%)	0.856^c^
	Female	32 (53.3%)	30 (50.8%)	
BMI (kg/m^2^)	Median (range)	22.3 (14.4–32.4)	23.2 (15.0–35.3)	0.132^b^
WHO-PS	≥ 1	18 (30.0%)	13 (22.0%)	0.404^c^
	0	42 (70.0%)	46 (78.0%)	
Compromised host	Yes	26 (43.3%)	14 (23.7%)	0.033*^c^
	No	34 (56.7%)	45 (76.3%)	
Smoking	Yes	19 (31.7%)	21 (35.6%)	0.701^c^
	No	41 (68.3%)	38 (64.4%)	
CT–SP	Yes	34 (56.7%)	18 (30.5%)	0.006*^c^
	No	26 (43.3%)	41 (69.5%)	
SMI (cm^2^/m^2^)	Median (range)	37.1 (21.1–57.7)	44.3 (21.4–63.0)	0.020*^b^
Alb (g/dL)^a^	Median (range)	3.4 (2.2–4.4)	4.0 (2.1–4.6)	< 0.001*^b^
Hospitalization duration (days)	Median (range)	12.0 (5–60)	8.0 (4–23)	< 0.001*^b^

A total number in each variable is 119 except for:

^a^*n* = 88, 5 patients in severe DNIs group and 26 patients in mild DNIs group had missing data

^b^Mann–Whitney *U* test

^c^Fisher’s exact test

^d^Chi-squared test.

*p < 0.05

*BMI* Body mass index, *WHO-PS* World health organization performance status, *CT–SP* Computed tomography-assessed sarcopenia, *SMI* Skeletal muscle index, *CRP* C-reactive protein, *WBC* White blood cell

### Treatments and clinical outcomes

All patients in the severe DNI group required surgical intervention, including incisional drainage and debridement of necrotic tissues. As expected, both the duration of intravenous antibiotic therapy and the length of hospitalization were significantly longer for patients with severe DNIs. The most common initial empirical antibiotic regimen in the severe DNI group was a combination of sulbactam/ampicillin and clindamycin, whereas the mild DNI group was predominantly treated with SBT/ABPC monotherapy (data not shown). These initial regimens accounted for approximately 95% of all cases and were subsequently adjusted based on antimicrobial susceptibility test results.

### Multivariable and correlation analyses

**Fig. 2:**
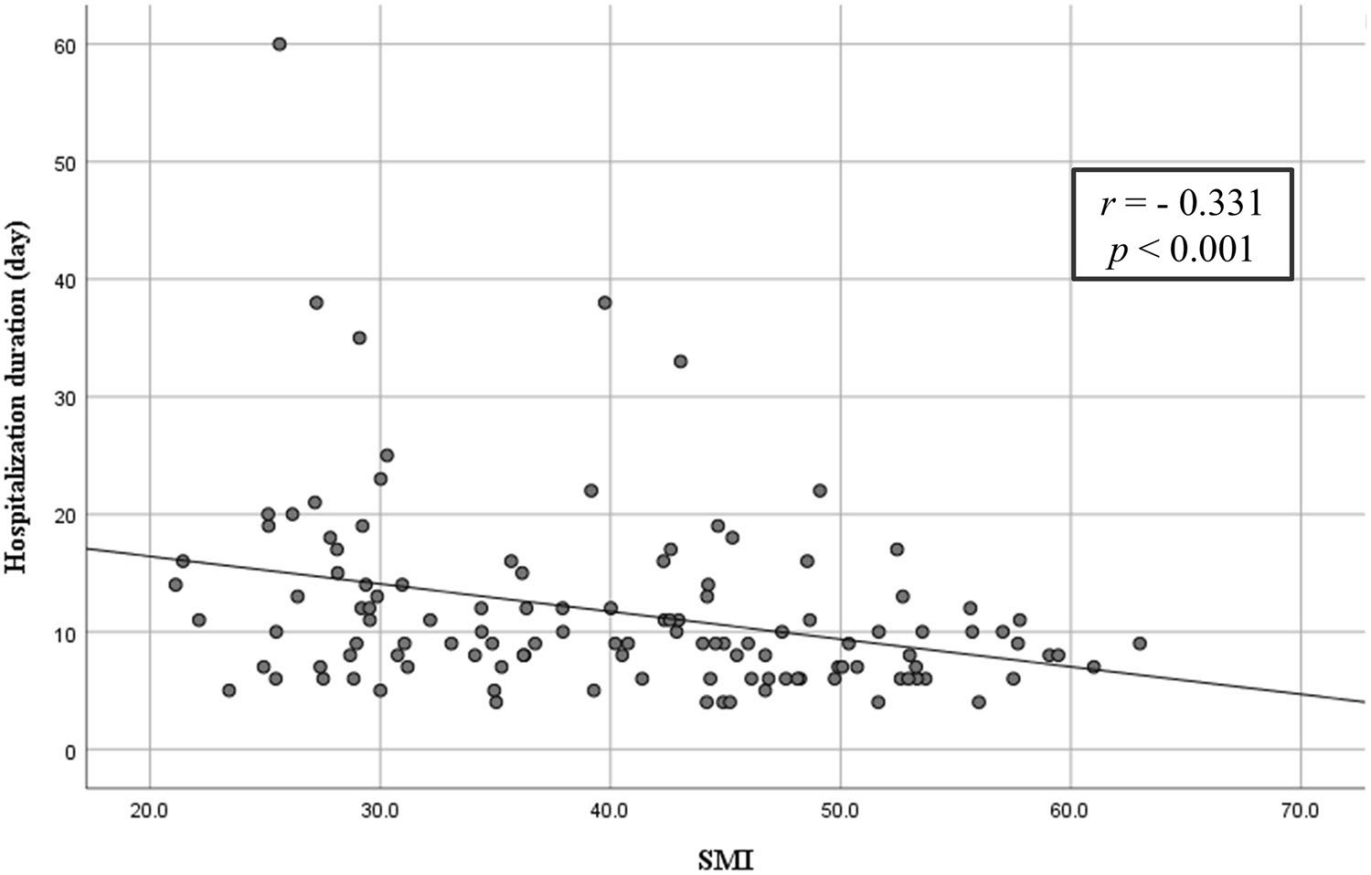
Correlation between SMI and the hospitalization duration. A significant negative correlation was observed between SMI and hospitalization duration, as calculated using Spearman’s rank correlation coefficient (*r* =− 0.331, *p* < 0.001)

Prior to performing multivariable logistic regression, an assessment for multicollinearity was conducted. The VIF was below 1.6 for all variables, indicating no significant collinearity and allowing all selected variables to be retained in the model. The multivariable analysis revealed that only CT–SP was an independent risk factor associated with severe odontogenic DNIs (Table 2). The Hosmer–Lemeshow test indicated a good model fit (*p* = 0.891). Finally, the relationship between SMI and the hospitalization duration was examined using Spearman’s rank correlation analysis. This revealed a statistically significant, weak negative correlation between the two variables (*r* = − 0.331, *p* < 0.001), as depicted in Fig. 2.

**Table 2:** Results of the multivariate logistic regression analysis of the risk factors for severe DNIs

Variable	*p* value	Odds ratio	95% CI	
			Lower	Upper
WHO-PS ≥ 1 (vs. 0)	0.244	0.517	0.170	1.570
Compromised host (vs. No)	0.158	1.959	0.770	4.983
Smoking (vs. No)	0.888	0.943	0.415	2.142
CT–SP (vs. No)	0.019*	3.041	1.199	7.714

*p < 0.05

*WHO-PS* World health organization performance status, *CT–SP* Computed tomography-assessed sarcopenia

## Discussion

This study aimed to investigate the relationship between CT–SP and the severity and hospitalization duration of odontogenic DNIs. Of 119 patients, 60 (50.4%) had severe odontogenic DNIs. Multivariate analysis showed that CT–SP (OR = 3.04) was an independent risk factor for severe odontogenic DNIs. In addition, Spearman’s rank correlation coefficient was *r* =− 0.331 (*p* < 0.001), indicating a weak negative correlation, suggesting that diminished muscle mass is associated not only with a more severe initial presentation but also with a prolonged recovery. A key finding of our multivariate analysis was that CT–SP emerged as a significant independent risk factor, whereas the 'Compromised host' status, despite including conditions like diabetes, did not. This suggests that sarcopenia, as a direct measure of physiological frailty, may be a more potent indicator of a patient’s diminished reserve than the mere presence of a chronic disease diagnosis.

The association between sarcopenia and severe DNIs is likely multifactorial, explained by a vicious cycle involving frailty, inflammation, and malnutrition. Sarcopenia is a cornerstone of frailty, which is often associated with a reduced capacity for self-care, including daily oral hygiene [23, 24]. This decline in oral self-care can lead to compromised oral health, allowing otherwise manageable dental conditions to progress into severe infections. This cumulative deterioration in oral health, which itself is linked to subsequent physical and cognitive decline, is defined as 'oral frailty' and serves as a crucial indicator of systemic vulnerability [25]. Thus, sarcopenia-associated frailty may precipitate severe DNIs through the pathway of deteriorating oral health. Skeletal muscle is the body's most important reservoir of proteins and a repository of amino acids for systemic needs, such as during starvation [26, 27]. In inflammatory states like cancer, cytokines drive muscle wasting by stimulating protein catabolism and suppressing its synthesis [14, 28]. Furthermore, certain amino acids, including glutamine, are known to modulate immune cells [29]. Therefore, the reduction in muscle mass may impair the host's infection defense capability and promote rapid progression to DNI or necrotizing fasciitis. Conversely, a severe DNI is a potent stimulus for a systemic inflammatory response, as shown by significantly higher CRP levels in the severe group. This systemic inflammation, driven by pro-inflammatory cytokines, such as interleukin-6, tumor necrosis factor-α, and transforming growth factor-β, promotes muscle catabolism, leading to an accelerated loss of muscle mass which can subsequently worsen the underlying inflammatory condition [14, 30]. Malnutrition acts as a critical intermediary in this cycle. A baseline state of malnutrition may exist in sarcopenic individuals, and the acute onset of a DNI with associated dysphagia and odynophagia precipitates a rapid decline in nutritional status. Notably, while serum albumin levels were significantly lower in the severe DNI group in univariate analysis (*p* < 0.001), this variable was not included in the final multivariate model due to a substantial proportion of missing data (26.1%). Although its exclusion was necessary to maintain the robustness of the regression model, this strong univariate association underscores the critical interplay between nutritional status and sarcopenia in this patient cohort. This is also consistent with findings from a related study, where the PNI—an index incorporating albumin—predicted prolonged hospitalization [9]. A recent study by Ohyama et al. reported a correlation between CT–SP, the PNI, and poor prognosis in patients with oral squamous cell carcinoma [15]. The present study established a direct correlation between CT–SP and the severity DNIs. Taken together with our previous research [9], these findings advocate for an integrated conceptual framework—what we term the 'Frailty–Inflammation–Malnutrition' axis—as a plausible explanatory model for the pathophysiology of severe DNIs. This concept underscores the necessity of addressing these interconnected factors not in isolation, but concurrently, to improve patient outcomes. Therefore, our findings suggest a compelling rationale for integrating opportunistic screening for low skeletal muscle mass (CT–SP) into the routine radiological workup for patients with head and neck infections. A low SMI, derived from a standard cervical CT scan, may serve as a potential "frailty biomarker." This would flag high-risk individuals who may warrant comprehensive geriatric assessment, targeted nutritional support, and more intensive clinical surveillance.

To the best of our knowledge, this is the first study to investigate the association between CT–SP and odontogenic DNIs. Our primary finding is that low skeletal muscle mass, as defined by CT–SP, correlates significantly with both the severity and the hospitalization duration of odontogenic DNIs. However, these findings must be interpreted in the context of several limitations. First, this study has limitations inherent to its retrospective, single-center design, which may have introduced selection bias. Furthermore, we assessed only skeletal muscle mass via CT and did not perform functional assessments (e.g., grip strength, gait speed) required for a definitive diagnosis of sarcopenia. Therefore, our findings specifically relate to CT-assessed low muscle mass, rather than clinically confirmed sarcopenia. In addition, the cross-sectional nature of our study design precludes any inference of causality. While we observed a strong association, we cannot rule out the possibility of reverse causality; that is, a severe DNI itself constitutes a significant catabolic stressor that can induce acute muscle loss, potentially confounding the association with a pre-existing state of low muscle mass. Furthermore, the broad age range of our cohort makes it difficult to distinguish between primary (age-related) and secondary (disease-related) low muscle mass. Pre-existing low muscle mass in older adults and acute muscle wasting induced by the infection in younger patients could not be differentiated, which is another limitation of the current study design. Second, another important consideration is the necessary exclusion of serum albumin from our multivariable model, which was unavoidable due to a high proportion of missing data (26.1%). Given its strong univariate association with severe DNIs, the absence of albumin as a potential confounding factor may have artificially inflated the apparent predictive significance of CT–SP. This potential for bias requires cautious interpretation of our results. Third, this study did not incorporate a direct, standardized assessment of oral hygiene practices or periodontal status. While our study's primary aim was to investigate a systemic host factor, the absence of these local data—which are the direct etiological source of the infection—is a limitation stemming from the retrospective reliance on medical records that often lack such detailed dental information. Finally, CT-based assessment of skeletal muscle mass assessment has constraints, and the optimal diagnostic threshold remains debatable, potentially varying among ethnic groups [31, 32]. Therefore, future large-scale, prospective, multicenter studies that incorporate functional assessments—such as grip strength and gait speed—are warranted to provide a more comprehensive understanding of sarcopenia’s role in DNI outcomes.

In conclusion, this study provides novel evidence that CT–SP is a significant risk factor associated with increased severity of odontogenic DNIs. These findings may imply that diligent oral health management and surveillance could be particularly crucial in patients with low skeletal muscle mass (as identified by CT–SP) to mitigate the risk of developing severe, potentially life-threatening, DNIs. Future studies are needed to confirm the direct impact of oral hygiene status on this population.

## Data Availability

The data that support the findings of this study are available from the corresponding author upon reasonable request.
